# Re-examining the reliability and validity of 30-15_IFT_ for VO_2_max prediction in male collegiate soccer players: a pilot study

**DOI:** 10.3389/fphys.2025.1668250

**Published:** 2025-10-17

**Authors:** Ruiqi Cheng, Weian Lin, Lin Song, Jinchen Pan, Ning Wang, Xiaotian Li

**Affiliations:** ^1^ School of Athletic Performance, Shanghai University of Sport, Shanghai, China; ^2^ The Key Laboratory of Adolescent Health Assessment and Exercise Intervention of the Ministry of Education, East China Normal University, Shanghai, China; ^3^ China Basketball College, Beijing Sport University, Beijing, China; ^4^ Department of Physical Education and Research, Central South University, Changsha, China; ^5^ School of Sport Training, Wuhan Sports University, Wuhan, China

**Keywords:** field test, change of direction, between-efforts recovery, anaerobic capacity, aerobic capacity

## Abstract

**Purpose:**

This pilot study aimed to determine the reliability and validity of the 30-15 Intermittent Fitness Test (30-15_IFT_) in male collegiate soccer players. A secondary aim was to develop a new, population-specific equation for predicting maximal oxygen uptake (VO_2_max) and to compare its predictive validity against a widely used general equation.

**Methods:**

Twenty well-trained male collegiate soccer players (age 19.5 ± 1.3 years, height 177.8 ± 6.3 cm, body mass 68.0 ± 14.3 kg; training experience 10.8 ± 3.0 years) participated in this study, and goalkeepers and players with injuries were excluded. A repeated-measures design was utilized. The participants completed three testing sessions separated by 1-week intervals: one trial of a continuous treadmill running test (CT) with running speed increasing by 1 km/h every minute to assess the validity of the 30-15_IFT_ and two trials of the 30-15_IFT_ to evaluate reliability. The 30-15_IFT_ involves 30-s runs across a 40-m course interspersed with 15 s of walking, with running speed increasing by 0.5 km/h every 45-s stage. Maximal intermittent running velocity (V_IFT_), maximum heart rate (HR_max_), and maximal oxygen consumption (VO_2_max) were collected for both tests. Reliability was assessed using the intraclass correlation coefficient (ICC) and typical error (TE). Validity was evaluated via Pearson correlation and Bland-Altman analysis. A multiple linear regression model was developed and cross-validated, with its predictive accuracy and agreement directly compared to those of the equation.

**Results:**

The 30-15_IFT_ demonstrated high reliability for all key metrics (ICC = 0.81–0.92, CV = 1.43–1.69%). Despite large correlations with CT measures (r = 0.62–0.77), Bland-Altman analysis revealed significant systematic bias and wide limits of agreement. The newly developed population-specific equation (r = 0.72, SEE = 2.90 mL/kg/min) demonstrated substantially lower bias (SEE = 2.90 mL/kg/min) compared to the general equation when applied to this cohort (SEE = 4.91 mL/kg/min).

**Conclusion:**

This pilot study demonstrates that the 30-15_IFT_ is a reliable tool for monitoring sport-specific performance, but should not be used interchangeably with laboratory-based tests due to significant disagreement. The application of general prediction equations can lead to considerable error. Future research should therefore focus on developing and validating these prediction models in larger, more diverse populations to improve their predictive accuracy and generalizability.

## 1 Introduction

Soccer is a typical intermittent team sport characterized by repeated high-intensity activities, including sprints, accelerations, decelerations, and rapid changes of direction (Carling et al., 2012). Due to the frequent occurrence of high-intensity movements during matches, players are required to repeatedly perform such efforts during critical phases, while maintaining a high level of aerobic endurance to support intensity regulation and recovery throughout the game (Harper et al., 2019). Notably, both the duration and repeatability of high-intensity running are strongly correlated with an athlete’s aerobic capacity (VO_2_max), with this relationship becoming particularly pronounced during the latter stages of the game—especially in the final 15 min (Gharbi et al., 2015; Grgic et al., 2019). Therefore, the accurate assessment of a player’s aerobic capacity is a cornerstone of effective physical preparation in soccer.

Incremental laboratory-based tests using treadmills or cycling ergometers are widely employed to assess cardiorespiratory fitness. However, these tests are time-consuming, require expensive equipment, and often interfere with athletes’ regular training schedules due to the need for repeated laboratory visits (Bassett and Howley, 2000). As a result, indirect assessment methods have gained increasing attention as practical alternatives (Flouris et al., 2010). To address the limitations of conventional lab-based assessments in athletic contexts, Buchheit and colleagues developed the 30-15 Intermittent Fitness Test (30-15_IFT_), a field-based test with greater ecological validity (Buchheit, 2009). The 30-15_IFT_ is an intermittent, incremental shuttle run test incorporating change-of-direction movements, enabling simultaneous evaluation of aerobic and anaerobic fitness, inter-effort recovery capacity, anaerobic speed reserve, and change-of-direction ability (Jeličić et al., 2020). Recent studies have confirmed the high reliability of the 30-15_IFT_ across various team sports, including handball, basketball, football, ice hockey, and rugby (Impellizzeri and Marcora, 2009; Jeličić et al., 2020; Grgic et al., 2021). One of the key advantages of the 30-15_IFT_ lies in its output variable—the maximal intermittent running speed (V_IFT_)—which can be used to prescribe individualized high-intensity interval training (HIIT) programs. This feature effectively overcomes the limitations of traditional continuous tests, which often fail to capture the sport-specific demands of match play (Čović et al., 2016). Moreover, HIIT prescriptions based on V_IFT_ have been shown to significantly reduce inter-individual variability in training intensity within teams (with a coefficient of variation [CV] of approximately 3%), thereby enhancing the homogeneity and standardization of group training sessions (Flouris et al., 2010). This provides a more effective strategy for implementing precise physical conditioning interventions in sports science practice.

Although the 30-15_IFT_ offers a strong alignment with the physiological and movement demands of team sports, its capacity to predict VO_2_max remains significantly different from that of gold standard measurements (ES = 0.84–1.10) (Čović et al., 2016; Jeličić et al., 2020). The estimated VO_2_max from the 30-15_IFT_ (VO_2_max-IFT) was calculated using the formula established by Buchheit (2008), which was based on a sample of 59 youth athletes (age, 16.2 ± 2.3 years). The validity of applying such a specific equation universally is questionable, as cardiorespiratory fitness is influenced by a multitude of factors, including sex, ethnicity, training status, and lifestyle (Pandey et al., 2016). This issue of generalizability is compounded by a broader sample bias in the existing literature, which has predominantly focused on professional or youth athletes (Stanković et al., 2021). Compared with youth or professional athletes, collegiate athletes exhibit distinct methodological and practical characteristics. On the one hand, they are typically in a semi-professional state, required to participate in high-level competitions while lacking systematic training, monitoring, and rehabilitation resources. On the other hand, their training load and recovery conditions are often markedly constrained by academic commitments, resulting in greater variability in athletic performance and fitness assessment outcomes (Bozzini et al., 2020). Furthermore, collegiate athletes are generally more biologically mature and no longer display the typical growth and developmental characteristics of youth academy players, a distinction that may influence their fitness adaptations and training responses (Gundersen et al., 2025). In contrast, youth athletes are largely managed within centralized academy systems, while professional athletes benefit from well-established training and medical support structures (McFadden et al., 2023). Therefore, focusing on collegiate athletes not only addresses a critical gap in the existing literature but also provides evidence-based insights for training monitoring and fitness evaluation in both collegiate and semi-professional populations.

Therefore, the primary purpose of this pilot study was to evaluate the reliability and validity of the 30-15_IFT_ for VO_2_max prediction in male collegiate soccer players. A secondary aim was to develop a new, population-specific prediction equation for VO_2_max and to directly compare its predictive validity against the Buchheit (2008) formula. We hypothesized that while the 30-15_IFT_ would be reliable, the new population-specific equation would demonstrate superior accuracy and agreement compared to the general formula.

## 2 Methods

### 2.1 Participants

Twenty male well-trained collegiate soccer players (Table 1) volunteered to participate in this study. All participants are from the Central South University soccer team and have performed well in the China University Football Association (CUFA), winning multiple provincial championships. The players trained 5.5 ± 1.2 times per week (11.8 ± 2.1 h per week) and testing took place during the competitive season. Goalkeepers were excluded due to aerobic capacity differences in soccer positions (Nobari et al., 2021). All players were free from cardiovascular or respiratory disease and had no injuries at the time of testing. The study was approved by the Ethics Committee of the Wuhan Sport University according to the Helsinki Declaration guidelines. Participants were fully informed and signed a consent form that indicated they could withdraw from the study at any time.

**TABLE 1:** Physical characteristics of subjects (*n* = 20).

Variables	Mean ± SD
Age (years)	19.5 ± 1.3
Height (cm)	177.8 ± 6.3
Body mass (kg)	68.0 ± 14.3
Training experience (years)	10.8 ± 3.0

### 2.2 Design

**FIGURE 1:**
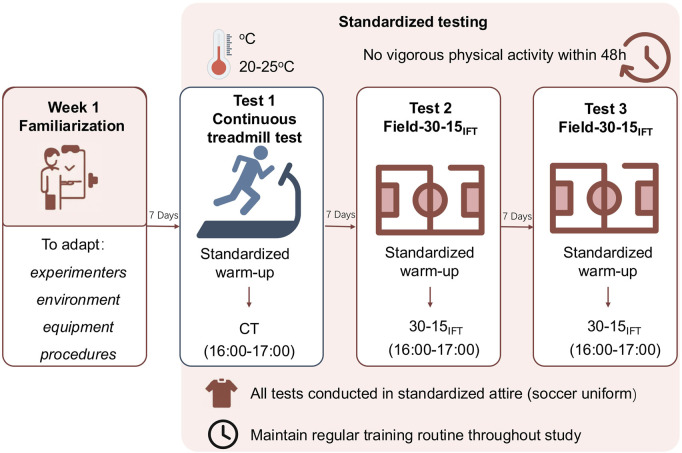
Simplified experimental protocol.

This study employed a repeated-measures design. A week before the main experimental protocol, participants had one habituation session to familiarize themselves with the experimenter, laboratory, materials, and exercise test to minimize the learning effect and ensure exercise test reliability. All three test sessions were conducted at the same time of day (between 4:00 p.m. and 5:00 p.m.), with a 7-day interval between each session. The first session was conducted in a laboratory setting using a motorized treadmill to determine maximal oxygen uptake (VO_2_max) and maximal heart rate (HRmax) as reference measures. The second and third sessions were conducted on an outdoor grass field where the participants normally trained, using the 30-15 Intermittent Fitness Test (30-15_IFT_) as the test protocol. On each testing day, participants wore their standard soccer match apparel and completed a standardized warm-up before the 30-15_IFT_, which included 5–10 min of moderate-intensity jogging followed by 5 min of static and dynamic stretching. To minimize the impact of fatigue, all participants were instructed to refrain from engaging in any vigorous physical activity for at least 48 h before each test session. Throughout the study period, participants maintained their regular training routines. All tests were performed under similar environmental conditions, with ambient temperatures ranging from 20 °C to 25 °C, to ensure consistency across sessions. The simplified experimental protocol is shown in Figure 1.

### 2.3 Continuous incremental running test on treadmill

All participants performed a continuous treadmill test (CT) in a controlled laboratory environment (ambient temperature ∼25 °C). The test was conducted on a motorized treadmill (Cosmed, Rome, Italy) with the incline set at 1°. Prior to testing, participants completed a standardized dynamic warm-up targeting the lower limbs, including leg swings, walking lunges, lateral lunges, ankle hops, and single-leg hops. The test protocol began at a speed of 5 km/h, with speed increasing by 1 km/h every minute until volitional exhaustion. Respiratory gas exchange data were collected in real time using a portable metabolic analyzer (K4b2, Cosmed, Rome), with recorded parameters including oxygen uptake (VO_2_), carbon dioxide production (VCO_2_), tidal volume (VT), minute ventilation (VE), respiratory exchange ratio (RER), and partial pressures of oxygen and carbon dioxide (PO_2_ and PCO_2_). All values were averaged over 5-s intervals. Maximal oxygen uptake in CT (VO_2_max-CT) was defined as the highest average VO_2_ observed over any four consecutive 20-s intervals during the test. Heart rate was continuously monitored at a frequency of 1 Hz using a Polar heart rate monitor (Polar, Finland), and the heart rate corresponding to VO_2_max-CT was recorded as the maximal heart rate (HRmax-CT). The final treadmill speed achieved at the point of VO_2_max-CT was recorded as the maximal treadmill velocity (V_CT_). Before each test, the gas analysis system was calibrated according to the manufacturer’s instructions to ensure measurement accuracy.

### 2.4 The 30-15 Intermittent Fitness Test

**FIGURE 2:**
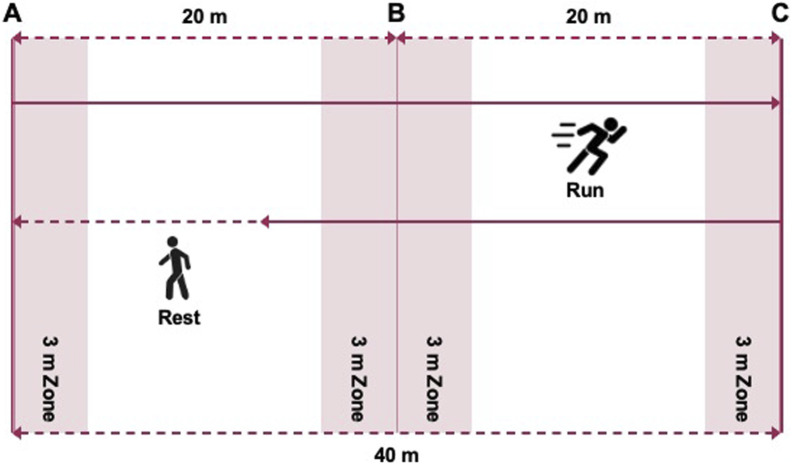
The course design for the 30-15 Intermittent Fitness Test.

The 30-15 Intermittent Fitness Test (30-15_IFT_) was administered according to the protocol described by Buchheit (2008). The course design for the 30-15_IFT_ is shown in Figure 2. The test consists of alternating 30-s shuttle runs and 15-s passive recovery periods. Participants followed a pre-recorded audio cue (APP: 30-15IFT) and started running from marker line A at 8 km/h, increasing the speed by 0.5 km/h per level. They ran back and forth between two lines 40 m apart at a certain speed during the 30 s of exercise, followed by a 15-s recovery period to walk back to within the nearest 3-m zone and the nearest marker line (A/B/C), after which the next level of testing began. Participants were encouraged to complete as many stages as possible. The test was terminated when any of the following criteria were met: (1) voluntary cessation by the participant, or (2) failure to reach the 3-m buffer zone before the audio signal on three consecutive occasions. The final completed stage speed was recorded as the participant’s maximal intermittent running speed (V_IFT_). Maximal oxygen uptake in 30-15_IFT_ (VO_2_max-IFT, in ml·min^-1^·kg^-1^) was estimated using Buchheit’s predictive equation (2008): VO_2_max-IFT = 28.3–(2.15 × gender)–(0.741 × age)–(0.0357 × body mass) + (0.0586 × age × V_IFT_) + (1.03 × V_IFT_) where gender was coded as 1 for males and 2 for females. Participants’ heart rates in 30-15_IFT_ (HR_max_-IFT) were measured throughout the 30-15_IFT_ using the Polar Team Pro System (Polar Team Pro System, Polar Electro, Kempele, Finland).

### 2.5 Statistical analyses

Data were presented as either mean ± SD or mean with 95% confidence intervals (95% CI) where specified. Normality of data was assessed by the Shapiro-Wilk statistic, and homogeneity of variance was verified with the Levene test. Reliability of the 30-15_IFT_ was examined using the intraclass correlation coefficient (ICC), typical error of measurement (TE) expressed as a coefficient of variation (CV), and smallest worthwhile change (SWC) (Atkinson and Nevill, 1998). To assess the magnitude of the ICC, the threshold values were 0.1, 0.3, 0.5, 0.7, 0.9, and 1.0 for low, moderate, high, very high, nearly perfect, and perfect, respectively (Koo and Li, 2016). The SWC was calculated as 0.2 × between-subjects SD. In line with previous research, if the TE was higher than the SWC, the evaluation of the test was marginal; if the TE was similar to the SWC, the evaluation was “OK”; and if the TE was less than the SWC, an evaluation of “good” was given to the test.

The validity between maximal oxygen uptake (VO_2_max), maximum heart rate (HR_max_), and the End-running velocity of 30-15_IFT_ and CT was assessed using Pearson correlation (r), and Spearman correlation was used when the data did not conform to a normal distribution. Correlation values denoted association between variables and tests as small (r = 0.1–0.3), moderate (r = 0.3–0.5), large (r = 0.5–0.7), very large (r = 0.7–0.9), and almost perfect (r = 0.9–1.0) (Hopkins et al., 2009; Jeličić et al., 2020). The practical significance of differences between consecutive trials and the magnitude of differences between 30-15_IFT_ and CT were also expressed as standardized mean differences (Cohen’s effect size; ES). ESs were classified as trivial (<0.19), small (0.20–0.59), moderate (0.60–1.19), large (1.20–1.99), and very large (2.0–4.0) (Hopkins et al., 2009). Furthermore, a multiple linear regression model was used to establish the link between VO_2_max-CT and all the variables in Buchheit’s equation. We excluded the Age * V_IFT_ interaction term, as it is primarily relevant for developing adolescents and was not a significant predictor within the narrow age range of our collegiate sample. To compare the validity of two equations, Pearson’s r was used to assess the correlation strength, and the standard error of estimate (SEE) and Bland-Altman analysis were used to analyze the prediction errors. All the statistical analyses were performed using R, version 4.4.2 (R Core Team, Vienna, Austria; https://www.R-project.org). The statistical significance level was set at p < 0.05.

## 3 Results

### 3.1 Reliability

The test-retest reliability statistics obtained during the 30-15_IFT_ are shown in Table 2. There were no significant differences in the estimated VO_2_max-IFT (p = 0.12, ES = 0.36), HR_max_-IFT (p = 0.37, ES = 0.21), and V_IFT_ (p = 0.13, ES = 0.35) between the test-retest trials. The reliability ratings for VO_2_max-IFT (ICC = 0.91, CV = 1.43%), HR_max_-IFT (ICC = 0.81, CV = 1.46%), and V_IFT_ (ICC = 0.92, CV = 1.69%) were high and very high between the two trials. The TE results (VO_2_max-IFT = 0.77; HR_max_-IFT = 2.76; V_IFT_ = 0.35) were higher than SWC (VO_2_max-IFT = 0.56 mL/kg/min; HR_max_-IFT = 1.28 bpm; V_IFT_ = 0.27 km/h), and their usefulness was evaluated as marginal.

**TABLE 2:** The test-retest reliability statistics for estimated maximal oxygen uptake (VO2max), end-running velocity (V_IFT_), and maximal heart rate (HRmax) during the 30-15 Intermittent Fitness Test in collegiate soccer players.

Variables	V_IFT_	VO_2_max	HR_max_
30-15IFT-1st trial	20.58 ± 1.36	53.85 ± 2.82	188.2 ± 6.39
30-15IFT-2nd trial	20.75 ± 1.07	54.25 ± 2.21	189.0 ± 6.39
T-Test (p)	0.13	0.12	0.37
Effect size	0.35	0.36	0.21
CV%	1.69	1.43	1.46
TE	0.35	0.77	2.76
SWC	0.27	0.56	1.28
ICC (95%CI)	0.92 (0.81, 0.97)	0.91 (0.7, 0.96)	0.81 (0.58, 0.92)
Effect size rating	Small	Small	Small
Usefulness rating	Marginal	Marginal	Marginal
Reliability rating	Nearly perfect	Nearly perfect	Very high

### 3.2 Validity

**FIGURE 3:**
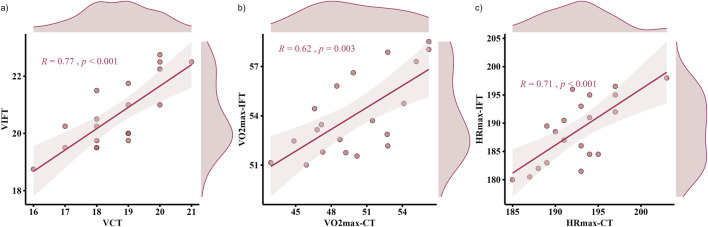
Pearson correlation between continuous running treadmill test (CT) and the 30-15_IFT_ for: **(a)** End-Running Velocity; **(b)** VO_2_max, and **(c)** HR_max_.

**FIGURE 4:**
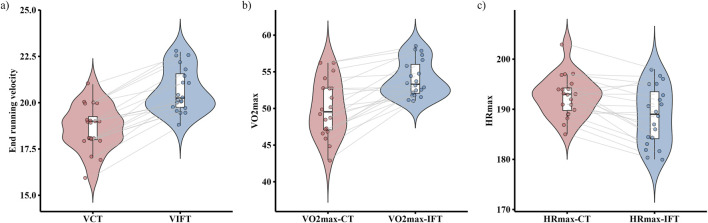
Differences between continuous running treadmill test (CT) and the 30-15_IFT_ for: **(a)** End-Running Velocity; **(b)** VO_2_max, and **(c)** HR_max_.

**FIGURE 5:**
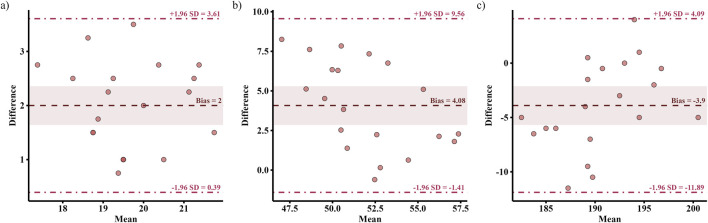
Bland-Altman plots with 95% limits of agreement between the continuous running treadmill test (CT) and the 30-15_IFT_ for: **(a)** End-Running Velocity; **(b)** VO_2_max, and **(c)** HR_max_; dashed lines represent 95% limits of agreement, and the shaded area represents the 95% confidence interval for the difference in means.

Figures 3–5 shows the results of the 30-15_IFT_ validity analyses for each indicator. The correlation analyses demonstrated criterion validity (Table 3), as evidenced by a large correlation between the 30-15_IFT_ and CT for VO_2_max (r = 0.62, p = 0.003) and a very large correlation between HR_max_ and end-running velocity (r = 0.71–0.77, p < 0.001). However, all variables in 30-15_IFT_ had moderate to very large differences compared to CT (ES = −0.96–2.44). This indicates that despite the strong correlations, a systematic bias exists, with the 30-15_IFT_ consistently overestimating physiological capacity and underestimating maximal heart rate relative to the gold-standard test.

**TABLE 3:** Pearson correlation coefficients for end-running velocity, maximal heart rate (HRmax), and maximal oxygen uptake (VO2max) during the continuous treadmill running test (CT) and average 30-15 Intermittent Fitness Test (30-15_IFT_) value.

Variables	CT	30-15_IFT_	ES	r	Correlation strength
End-running velocity (km/h)	18.65 ± 1.23	20.68 ± 1.18^***^	2.44	0.77	Very large
VO_2_max (mL/kg/min)	49.97 ± 3.85	54.05 ± 2.47^***^	1.46	0.62^¬^	Large
HR_max_ (bpm)	192.6 ± 4.15	188.6 ± 5.79^***^	−0.96	0.71	Very large

ES, effect size; bpm, beats per minute; * indicates statistically significant difference between CT and 30-15_IFT_ (**p < 0.01, ***p < 0.001); ^¬^indicates spearman correlation coefficient.

**FIGURE 6:**
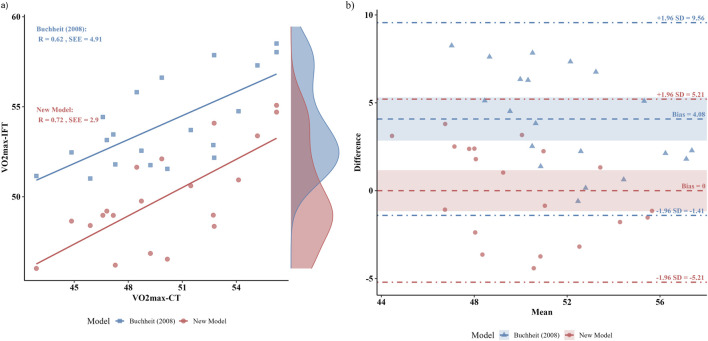
Comparative analysis of the predictive accuracy and agreement for the new population-specific model and the general Buchheit (2008) equation. **(a)** Predicted *versus* criterion VO2max. **(b)** Bland-Altman plots with 95% limits of agreement between the continuous running treadmill test (CT).

Bland-Altman plots (Figure 6) present the limits of agreement between the 30-15_IFT_ and the CT. The 30-15_IFT_ overestimated end-running velocity by 2.0 km/h (95% LoA: 0.39–3.61 km/h) and VO_2_max by 4.08 mL/kg/min (95% LoA: −1.41–9.56 mL/kg/min) compared to the CT. Conversely, HR_max_ was underestimated by an average of −3.9 bpm (95% LoA: −11.89 to 4.09 bpm).

### 3.3 Multiple linear regression

For the 20 subjects, the VO2max-CT was significantly correlated with all variables and can be summarized by the following regression: VO2max-CT (mL/kg/min) = −8.85 + 2.35 * V_IFT_ + −0.13 * BM + 1 * Age (r = 0.72, p = 0.007, SEE = 2.90 mL/kg/min) (Figure 6a). The Bland-Altman plot (Figure 6b) shows that the new model exhibits little bias in VO_2_max (95% LoA: −5.21 to 5.21 mL/kg/min) compared to the CT.

## 4 Discussion

This pilot study aims to verify the reliability and validity of the 30-15 Intermittent Fitness Test (30-15_IFT_) in college soccer players and to explore whether the prediction of aerobic capacity requires different prediction formulas for various groups. The preliminary findings revealed that the 30-15_IFT_ demonstrates high test-retest reliability (ICC = 0.81–0.92, CV% = 1.43–1.69%) for V_IFT_, VO_2_max, and HR_max_. Although the 30-15_IFT_ showed a large to very large correlation with the gold-standard continuous treadmill test (CT), its validity is limited because of the bias (ES = 0.96–2.44). Our results also showed that the usefulness of the 30-15_IFT_ was marginal for all outcome measures. Furthermore, the novel, population-specific equation we developed for collegiate athletes yields a marked reduction in bias and enhanced predictive accuracy. These pilot findings critically highlight the inherent constraints of universal prediction models when applied to specific populations.

Reliability is a critical indicator for evaluating measurement error and is typically categorized into absolute reliability (i.e., the degree of variability in repeated measures for the same individual) and relative reliability (i.e., the consistency of an individual’s rank ordering within a group across repeated assessments) (de Vet et al., 2006; Impellizzeri and Marcora, 2009). Relative reliability is commonly assessed using the intraclass correlation coefficient (ICC), whereas absolute reliability is reflected by the coefficient of variation (CV) and typical error (TE). These metrics are of high practical value in both cross-sectional and longitudinal studies (de Vet et al., 2006). The present results showed high absolute reliability and relative reliability for V_IFT_, and higher than the standard (ICC>0.69 and CV<5%) set by Buchheit et al. (2011). No significant differences were observed between the two test trials—indicating stable test outcomes with no evidence of a learning effect (Paravlic et al., 2022). Our study showed similar reliability compared to previous studies targeting various athlete populations, including female basketball players (VO_2_max-IFT: ICC = 0.96, CV = 4.83%; V_IFT_: ICC = 0.85, CV = 5.99%) (Jeličić et al., 2020), futsal players (V_IFT_: ICC = 0.92–0.96, CV = 1.4–1.5%; HR_max_-IFT: ICC = 0.90–0.91, CV = 1.3–1.5%) (Valladares-Rodríguez et al., 2017), youth rugby players (V_IFT_: ICC = 0.89; HRmax: ICC = 0.96) (Scott et al., 2015), and female soccer players (VO_2_max-IFT: ICC = 0.94, CV = 1.6%; HR_max_-IFT: ICC = 0.96) (Jeličić et al., 2020). The high reliability of the 30-15_IFT_ observed in our study makes it comparable to other established field tests for soccer players, such as the widely used Yo-Yo Test, which has demonstrated similar reliability in previous studies (ICC: 0.78–0.98, CV: 3.7%–19.0%) (Grgic et al., 2019). In addition, Buchheit reported a strong correlation between performance on the 30-15_IFT_ and the Yo-Yo Intermittent Recovery Level 1 (IR1) test (r = 0.75; 90% CI = 0.57–0.86), indicating a degree of convergence between the two assessments. Although these tests may target slightly different physiological capacities, both exhibit similar sensitivity to training-induced changes (Stanković et al., 2021). Taken together, the 30-15_IFT_ can be regarded as a reliable and reproducible field-based tool for assessing aerobic fitness in collegiate male soccer players.

Although the continuous incremental treadmill test (CT) is widely regarded as the “gold standard” for evaluating aerobic endurance (Poole and Jones, 2017), its practical application is often constrained by environmental conditions, cost, time requirements, and technical complexity. In contrast, the present study established good criterion validity for the 30-15_IFT_. The primary outcome measures demonstrated large to very large correlations (r = 0.62–0.77) with their respective counterparts from the continuous treadmill test, supporting the practical utility of the 30-15_IFT_ for assessing aerobic fitness in collegiate male soccer players. Previous researches also support this opinion. Previous studies have shown similar results. Čović et al. reported moderate-to-strong linear correlations between the 30-15_IFT_ and CT in both VO_2_max (r = 0.67) and HR_max_ (r = 0.77) in elite female soccer players (Čović et al., 2016). Similar correlations (r = 0.69–0.74) were reported by Jeličić et al. between outcome measures taken during the 30-15_IFT_ and CT in female basketball players (Jeličić et al., 2020). Notably, while the strong correlations suggest the 30-15_IFT_ is a valid measurement, the question of whether it truly reflects an athlete’s capacity requires careful consideration. Our findings revealed a notable result: despite the strong relationship, the mean differences between the tests were practically significant, evidenced by moderate to very large effect sizes (ES = 0.96–2.44). Moreover, the Bland-Altman plots showed wide limits of agreement and a visible bias line. It indicated that the outcome measures of the 30-15_IFT_ have large random error, limiting the utility of the 30-15_IFT_ as a direct proxy for true aerobic capacity. Previous studies have also reported this significant difference in various populations (female basketball players: ES (d) = 0.84–3.23; female soccer players: ES (d) = 0.98–1.60; Infantry members: ES (η^2^) = 0.158–0.623) (Čović et al., 2016; Jeličić et al., 2020; Paravlic et al., 2022). On the one hand, previous studies have reported a typical difference of 2–5 km/h between the two tests and have been confirmed in multiple empirical studies (Buchheit et al., 2010; Jeličić et al., 2020). The higher V_IFT_ values may be attributed to the protocol of the 30-15_IFT_, as its intermittent structure and constant 180° changes of direction place significant demands on both anaerobic capacity and the ability to efficiently change direction (Darrall-Jones et al., 2015; Scott et al., 2015). In this study, VO_2_max was estimated using Buchheit’s modified formula incorporating V_IFT_, age, and body mass. Results showed that the higher V_IFT_ values contributed to greater VO_2_max estimates. Furthermore, Jeličić et al. reported that a familiar training environment may better enable athletes to reach higher speeds compared to laboratory-based continuous treadmill running, leading to an overestimation of VO_2_max (Jeličić et al., 2020). In contrast, the lower HR_max_-IFT observed during the 30-15_IFT_ may be linked to the testing environment. The unfamiliar and restrictive nature of laboratory treadmill testing can induce psychological stress and elevate heart rate, whereas the familiar field setting of the 30-15_IFT_ may elicit a more ecologically valid physiological state (Buchheit, 2008; Paulsen et al., 2023). On the other hand, the observed differences between the two tests may lie in the limitations of applying a single, universal prediction equation to a demographically distinct population. The original Buchheit (2008) formula was developed on a different cohort, which may not accurately capture the specific characteristics of our sample. To investigate this, we tried to develop a population-specific model and directly compared its predictive performance against the Buchheit (2008) equation. The direct comparison of the models revealed that our equation exhibited a substantially lower systematic bias (Bias ≈0 vs. 4.08 mL/kg/min) and a smaller standard error of the estimate (SEE = 2.90 vs. 4.91 mL/kg/min). While this improved accuracy may be partly due to the model being calibrated for our specific and homogenous cohort of male collegiate soccer players, the observed reduction in prediction error highlights a key takeaway. It suggests that applying a general prediction equation to a specific population can lead to significant inaccuracies. Therefore, our findings strongly advocate for the development and use of population-specific models to enhance the precision of field-based physiological assessments.

In addition, the study evaluated the usefulness of the 30-15_IFT_ by comparing TE *versus* SWC. This comparison can help coaches conclude the significance of changes in performance due to training interventions or other factors. The results indicated that the usefulness of VO_2_max-IFT, HR_max_-IFT, and V_IFT_ was marginal with TE > SWC. However, for V_IFT_, TE at 0.35 km/h compared to SWC at 0.27 km/h resulted in a performance change of less than one phase of the exercise (±0.5 km/h). This suggests that an individual’s performance change of less than one phase (±0.5 km/h) can be considered ‘real and meaningful’. These findings suggest that while the 30-15_IFT_ should not be used as a direct substitute for laboratory-based assessment of maximal aerobic capacity, its high reliability and the usefulness in V_IFT_ make it an excellent and practical tool for monitoring meaningful longitudinal changes in sport-specific performance and assisting coaches in making informed decisions.

## 5 Limitations

While our study provides valuable insights into the reliability and validity of the 30-15_IFT_, several limitations should be acknowledged. First, the reliability was derived from only two trials. Although this is acceptable for calculating the ICC, more robust estimates of practically important values like typical error (TE) and the coefficient of variation (CV) are typically achieved with three or more trials (Hopkins, 2000). Second, our validity analysis, particularly the Bland-Altman plots, revealed wide LoA, suggesting that large random measurement error may limit the interchangeability of the tests for individual assessment. Future studies should consider using larger samples to better quantify this variability. The most significant limitation of this study is the small sample size (n = 20), which restricts the statistical power and generalizability of our findings.

As a pilot study, this work highlights several critical directions for future research. First, the proposed prediction equation requires external validation in a larger, more diverse cohort of collegiate athletes to establish its robustness and generalizability. Second, subsequent research should investigate whether similar predictive biases exist in other distinct populations, such as female collegiate athletes or those from different sports. Finally, future models could incorporate additional variables (e.g., anaerobic speed reserve or change-of-direction metrics) to determine if they further enhance the precision of VO_2_max prediction.

## 6 Conclusion

Final conclusions must be formulated carefully due to the small sample size. The 30-15 Intermittent Fitness Test is a reliable tool for assessing sport-specific fitness in male collegiate soccer players. Changes in V_IFT_ of less than one phase of the exercise (±0.5 km/h) are likely to represent a meaningful change in performance. However, due to significant systematic bias and large random error, it cannot be used interchangeably with continuous treadmill testing for assessing maximal aerobic capacity. Furthermore, this study demonstrates that general prediction equations for VO_2_max can introduce considerable error when applied to specific populations. Future research should therefore focus on developing and validating these prediction models in larger, more diverse populations to improve their predictive accuracy and generalizability.

## Data Availability

The original contributions presented in the study are included in the article/supplementary material, further inquiries can be directed to the corresponding authors.
